# Supplementary material for: An off-lattice discrete model to characterise filamentous yeast colony morphology
Source: PLoS Comput Biol. 2024 Nov 21;20(11):e1012605. doi: 10.1371/journal.pcbi.1012605 (PMC11620580; doi:10.1371/journal.pcbi.1012605)
Supplement: S1 Text — Table A. Effective sample size and acceptance threshold for ABC-MCMC results. ESS and ϵ acceptance threshold for ABC-MCMC algorithm. Each iteration of the chain is simulated up to the average pixel colony area of the respective colonies, allowing up to a 5% difference. In addition, the computation time for a single colony and their average cell number are given below. Computations are performed using an Intel Xeon CPU E5–2699 v3 (2.30GHz). Fig A. ABC-MCMC trace plots. Trace plots of ABC-MCMC algorithm for (a) AWRI 50 μm (ESS of 205.151), (b) AWRI 500 μm (ESS of 222.225) and (c) Simi White 50 μm (ESS of 251.980). Each chain is running until 20,000 iterations. Fig B. Individual colony simulations. Comparison between experiments and simulations with parameters inferred using individual colonies. (a–b) AWRI strain with 50 μm nutrient. (c–d) AWRI strain with 500 μm nutrient. (e) Simulation with θ = (n*, pa, psp, pps, γ) = (0.56, 0.10, 0.31, 0.65, 0.16) inferred S12 AWRI 50 μm experiments. (f) Simulation with θ = (n*, pa, psp, pps, γ) = (0.66, 0.10, 0.23, 0.54, 0.15) inferred S7 AWRI 50 μm experiments. (g) Simulation with θ = (n*, pa, psp, pps, γ) = (0.89, 0.09, 0.28, 0.67, 0.14) inferred S11 AWRI 500 μm experiments. (h) Simulation with θ = (n*, pa, psp, pps, γ) = (0.78, 0.08, 0.20, 0.70, 0.13) inferred S8 AWRI 500 μm experiments. Fig C. ABC-MCMC trace plot with uninformative priors. Trace plot of ABC-MCMC algorithm for AWRI 796 50 μm using uniform priors. (PDF) [file pcbi.1012605.s001.pdf]

Supplementary Material:
An off-lattice discrete model to characterise filamentous yeast colony morphology

Kai Li[1], J. Edward F. Green[1], Hayden Tronnolone[2], Alexander K. Y. Tam[3], Andrew J. Black[1], Jennifer M. Gardner[4], Joanna F. Sundstrom[4], Vladimir Jiranek[4,5,*], and Benjamin J. Binder[1]

[1]School of Computer and Mathematical Sciences, University of Adelaide, Adelaide SA, Australia

[2]College of Science and Engineering, Flinders University, Adelaide SA, Australia

[3]UniSA STEM, The University of South Australia, Mawson Lakes SA, Australia

[4]Discipline of Wine Science, Waite Campus, University of Adelaide, Urrbrae SA, Australia

[5]School of Biological Sciences, The University of Southampton, Southampton, United Kingdom

[*]Correspondence: V.Jiranek@soton.ac.uk

S1 Supplementary Material

ABC-MCMC Algorithm

All of the values used in the calibration of the ABC algorithm are listed in Table A. These include the reported effective sample size (ESS) values, ϵ threshold and colony areas. Furthermore, to assess how well the ABC-MCMC chain is mixing, we utilise trace plot analysis as shown in Figure A. These trace plots indicate sufficient mixing over the history of the chain.

Table A: ESS and ϵ acceptance threshold for ABC-MCMC algorithm. Each iteration of the chain is simulated up to the average pixel colony area of the respective colonies, allowing up to a 5% difference. In addition, the computation time for a single colony and their average cell number are given below. Computations are performed using an Intel Xeon CPU E5-2699 v3 (2.30GHz).

	ESS	ϵ	Area (pixels)	Compute time (s)	Cell number
AWRI 50 µM	205.15	0.030	573921	171	37599
AWRI 500 µM	222.23	0.010	1052348	401	81810
Simi White 50 µM	251.98	0.015	825329	324	54908

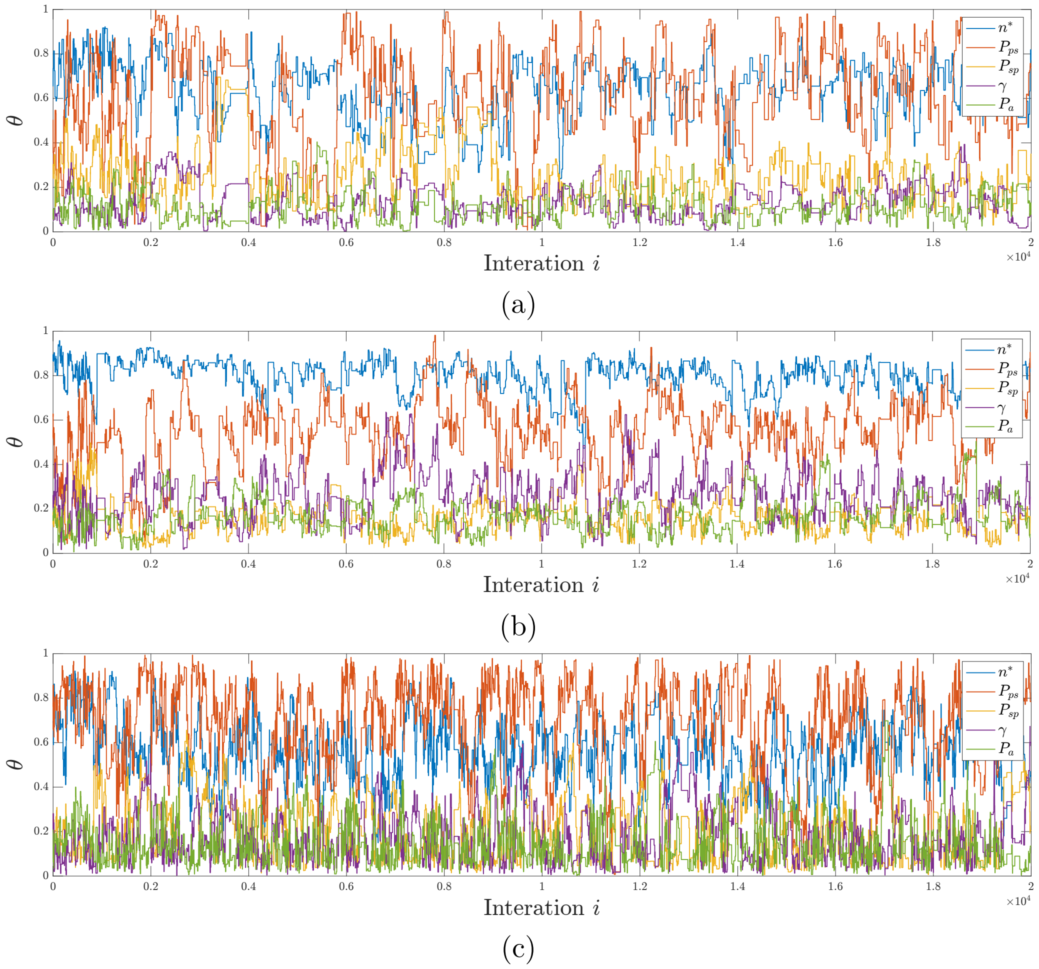

Figure A: Trace plots of ABC-MCMC algorithm for (a) AWRI 50 µM (ESS of 205.151), (b) AWRI 500 µM (ESS of 222.225) and (c) Simi White 50 µM (ESS of 251.980). Each chain is running until 20,000 iterations.

Parameter Inference for Individual Colonies

In this work, we presented results with parameter values inferred from the mean properties of multiple replicates of the same experimental conditions. Another possible approach to the inference is to infer parameters from each colony individually. To illustrate this, we selected two colonies from AWRI 50 µM and AWRI 500 µM and performed the inference as shown in Figure B. As expected, we can obtain very good qualitative agreement between the experimental and simulated colonies using this method. However, a disadvantage of this approach is the additional computational cost of inferring parameters on each image. Inferring parameters using the mean summary statistics was sufficient to differentiate between experimental conditions.

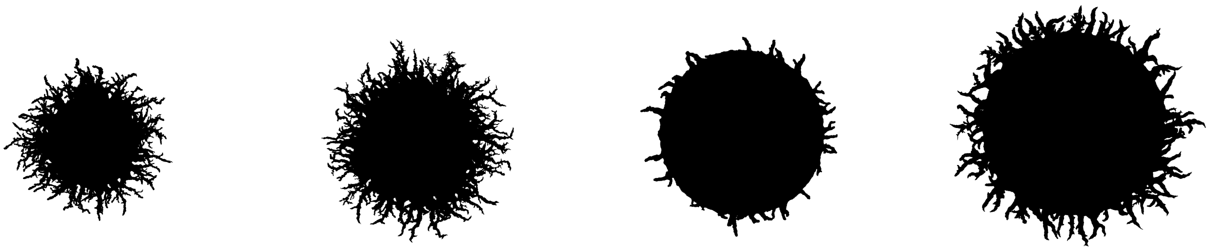

(a) AWRI 50 µM S12 experiment. (b) AWRI 50 µM S7 experiment. (c) AWRI 500 µM S11 experiment. (d) AWRI 500 µM S8 experiment.

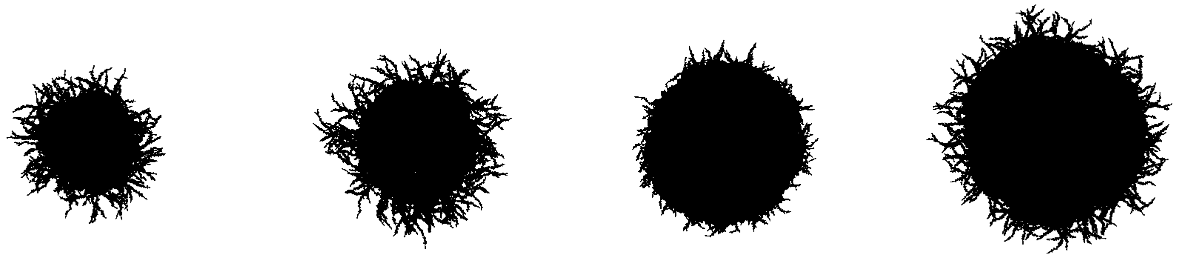

(e) Simulation (AWRI 50 µM) (f) Simulation (AWRI 50 µM) (g) Simulation (AWRI 500 µM) (h) Simulation (AWRI 500 µM)

Figure B: Comparison between experiments and simulations with parameters inferred using individual colonies. (a–b) AWRI strain with 50 µM nutrient. (c–d) AWRI strain with 500 µM nutrient. (e) Simulation with $\theta = (n^*, p_a, p_{sp}, p_{ps}, \gamma) = (0.56, 0.10, 0.31, 0.65, 0.16)$ inferred S12 AWRI 50 µM experiments. (f) Simulation with $\theta = (n^*, p_a, p_{sp}, p_{ps}, \gamma) = (0.66, 0.10, 0.23, 0.54, 0.15)$ inferred S7 AWRI 50 µM experiments. (g) Simulation with $\theta = (n^*, p_a, p_{sp}, p_{ps}, \gamma) = (0.89, 0.09, 0.28, 0.67, 0.14)$ inferred S11 AWRI 500 µM experiments. (h) Simulation with $\theta = (n^*, p_a, p_{sp}, p_{ps}, \gamma) = (0.78, 0.08, 0.20, 0.70, 0.13)$ inferred S8 AWRI 500 µM experiments.

Uninformative Priors

We also investigated the effect of priors on the system. To justify our use of informative Beta distribution priors, we compared our results to those using uninformative Uniform$(0, 1)$

priors with AWRI 50 µM experiments. The results of the uniform priors are given in Figure C. The mean values produced by informative priors in Fig. 10 were $\theta = (n^*, p_a, p_{sp}, p_{ps}, \gamma) = (0.67, 0.14, 0.25, 0.58, 0.12)$. In comparison, the mean values using the uniform priors were $\theta = (n^*, p_a, p_{sp}, p_{ps}, \gamma) = (0.61, 0.11, 0.22, 0.61, 0.12)$. The comparison shows our choice of priors did not have a large effect on resulting posteriors. The minor difference in the mean values could also be due to the shorter length of these chains, which are necessitated by the computational expense of the simulations.

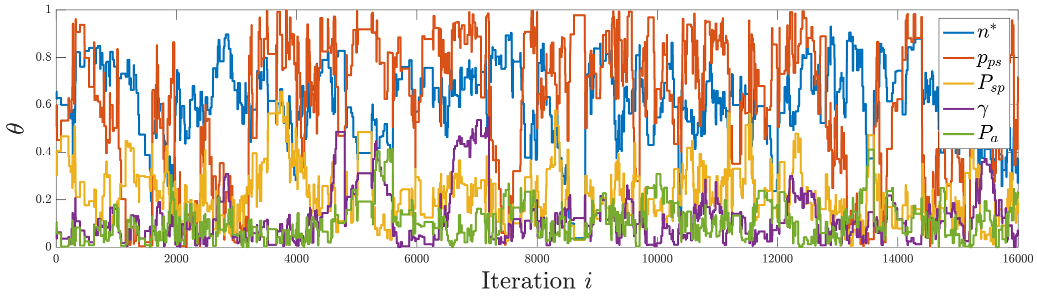

Figure C: Trace plot of ABC-MCMC algorithm for AWRI 796 50 µM using uniform priors.
