# Supplementary material for: An off-lattice discrete model to characterise filamentous yeast colony morphology
Source: PLoS Comput Biol. 2024 Nov 21;20(11):e1012605. doi: 10.1371/journal.pcbi.1012605 (PMC11620580; doi:10.1371/journal.pcbi.1012605)
Supplement: Attachment — Submitted filename: review-PCOMPBIOL-D-24-01245.pdf [file pcbi.1012605.s002.pdf]

Report on manuscript PCOMPBIOL-D-24-01245

The manuscript "An off-lattice discrete model to characterise filamentous yeast colony morphology" by Li et al. presents a mathematical model and corresponding statistical analysis for a model of two-dimensional yeast growth. The manuscript is overall very well written, and easy to follow. I would be happy to recommend the work for publication, however have several (relatively minor) concerns that I feel should be first addressed.

Specific comments:

1. My largest concern is relating to the claimed "nutrient limited transition" (or from other environmental stressors) from sated to pseudohyphal growth. However, the authors do not explicitly model nutrients or nuturient consumption. Furthermore, the authors also do not model crowding, rather specifying that the cell population switches from sated to pseudohyphal growth once a specific population/area is reached, based on the assumption that nutrient availability is a linearly decreasing function of cell population. This point is, I believe, critical, since the authors claim that the model parameters can distinguish between experiments of different nutrient concentrations (hence, I would expect nutrient *consumption* to be a critical factor).

   I cannot see why this later assumption would be the case in two-dimensional experiments, and this assumption should be justified (ideally through experimental validation). I understand that the authors do not, effectively, model time, however I would expect that the nutrient consumption depends not just on the growth rates but also parameters relating to the population composition (effectively, the p parameters). I would find the results more compelling if the transition was an emergent property rather than a pre-programmed behaviour.

2. Is competition (i.e., cell-pushing and other mechanical effects) known not to be important in the transition discussed in my previous comment? Furthermore, how do the cells regulate the transition as a "colony-level decision" (page 10)?
3. Further to my comment above, why do the authors not model time? Is time-series data simply not available for these experiments? In which case, how can the authors be sure that the model-experiment comparison is drawn at the correct "time"?
4. The authors should justify their choice of prior. The priors for p_{ps} and p_{sp} could reasonably be jusified as being uninformative, however the same cannot be said for the author parameters. How do the results depend on prior choice? Why do the authors not consider a uniform distribution (i.e., $\text{Beta}(1, 1)$)?
5. Did the authors consider performing a formal model-selection procesure for the three budding patterns proposed at the bottom of page 6?

Minor comments:

1. The authors should provide justification (or otherwise, a sensitivity-type analysis) for the parameters chosen in Table 2.1.
2. Do the authors expect any parameters to be identical between strains?
3. The authors state that it is possible to infer parameters for single experimental images, but this could lead to over fitting. I would expect, rather, to just have possible identifiability issues. However, if the summary statistics are comprensive enough and the variability in summary statistics small enough between images, the posteriors should be similar.
