# Supplementary material for: An off-lattice discrete model to characterise filamentous yeast colony morphology
Source: PLoS Comput Biol. 2024 Nov 21;20(11):e1012605. doi: 10.1371/journal.pcbi.1012605 (PMC11620580; doi:10.1371/journal.pcbi.1012605)
Supplement: Attachment — Submitted filename: Off_lattice_PLoS_Responses.pdf [file pcbi.1012605.s003.pdf]

Response to Reviewers

An off-lattice discrete model to characterise filamentous yeast colony morphology

Kai Li, J. Edward F. Green, Hayden Tronnolone, Alexander K. Y. Tam, Andrew J. Black, Jennifer M. Gardner, Joanna F. Sundstrom, Vladimir Jiranek, Benjamin J. Binder

Reviewer 1

The manuscript "An off-lattice discrete model to characterise filamentous yeast colony morphology" by Li et al. presents a mathematical model and corresponding statistical analysis for a model of two-dimensional yeast growth. The manuscript is overall very well written, and easy to follow. I would be happy to recommend the work for publication, however have several (relatively minor) concerns that I feel should be first addressed.

Response: We are grateful to Reviewer 1 for their careful reading of our manuscript and helpful suggestions. The changes we have made are outlined below, and printed in magenta within the paper for ease of identification.

Specific Comments

1. My largest concern is relating to the claimed "nutrient limited transition" (or from other environmental stressors) from sated to pseudohyphal growth. However, the authors do not explicitly model nutrients or nutrient consumption. Furthermore, the authors also do not model crowding, rather specifying that the cell population switches from sated to pseudohyphal growth once a specific population/area is reached, based on the assumption that nutrient availability is a linearly decreasing function of cell population. This point is, I believe, critical, since the authors claim that the model parameters can distinguish between experiments of different nutrient concentrations (hence, I would expect nutrient consumption to be a critical factor).

   I cannot see why this later assumption would be the case in two-dimensional experiments, and this assumption should be justified (ideally through experimental validation). I understand that the authors do not, effectively, model time, however I would expect that the nutrient consumption depends not just on the growth rates but also parameters relating to the population composition (effectively, the p parameters). I would find the results more compelling if the transition was an emergent property rather than a pre-programmed behaviour.

   Response: As stated in the manuscript, we did not model the nutrient concentration explicitly, but instead assumed that nutrient is a linearly decreasing function of cell

number. However, we acknowledge that we did not explicitly mention two more important pieces of information that help justify this assumption. Firstly, for simplicity we assume that all of the consumed nutrients are used for cell proliferation, as per previous modelling work [1]. It is also important to note that the cell size parameters given in Table 1 are known from the literature [2], and considered constant across strains and nutrient levels (§2.2, p. 7, Table 1). Assuming that the cells are ellipses in 2D (ellipsoids in 3D), then sated and pseudohyphal cells have areas/volumes that differ by only 1% (now mentioned in §2.2, p. 7, Lines 137–139). Thus, we assume that every cell requires the same amount of nutrients to proliferate regardless of whether it is sated or pseudohyphal, such that nutrient consumption does not depend on colony composition. Finally, experimental work [3] has shown that nutrient concentration in filamentous yeast colonies is spatially uniform. This fact, and the assumption that the amount of nutrient consumed per new cell is constant, guarantees that the nutrient concentration is a linearly decreasing function of cell number. Furthermore, although pseudohyphal growth is known to be triggered by low nutrient availability, the absolute nutrient concentration at which the transition occurs cannot be measured in experiments. Given that nutrient concentration decreases linearly with cell number, assuming that the cell number n^* governs the transition to pseudohyphal growth is equivalent to imposing a constant nutrient threshold whose magnitude is unknown, and may vary with strain and initial nutrient concentration. This is now explained in more detail in the manuscript (§2.2, p. 9, Lines 179–183).

In addition, as the reviewer states we do not explicitly model cell crowding. Instead, we implement partial volume exclusion whereby cell proliferation cannot occur if the centroid of the new cell would be inside the boundary of another cell. In experiments, cell growth is primarily confined to the 2D plane, such that colony area (viewed from above) is a good proxy for the number of cells in the experiment. This justifies using the cell number as a stopping criterion for the simulation.

2. Is competition (i.e., cell-pushing and other mechanical effects) known not to be important in the transition discussed in my previous comment? Furthermore, how do the cells regulate the transition as a "colony-level decision" (page 10)?

   Response: In the time since Gimeno et al. [2] described pseudohyphal growth, many studies have focused on isolating the genetic and signalling pathways that regulate pseudohyphal growth [4]. These pathways, for example short-chain alcohols acting as quorum sensing molecules, might also explain how the yeast colony regulates the cell shape transition as a colony level decision, which we now note in the manuscript (§2.2, p. 10, Lines 194–196).

   In contrast to these pathways, the effects of mechanics and cell pushing are less well-understood. Our aim was to develop a simple mathematical model based on cell shape changes. Decreasing computational complexity was an important consideration for our model, because the parameter inference methods require a large number of simulations. Neglecting mechanics also allowed us to focus on cell shape change alone, which was sufficient to yield results similar to experiments. Therefore, although competition might affect colony morphology, we expect its impact to be insignificant relative to the effect of cell shape changes. Our model neglects mechanics, but does incorporate cellular competition by partial volume exclusion, where we prevent a cell proliferation event from

occurring if the centre of the new cell would lie within the boundary of an existing cell. In practice, this imposes an upper limit on the local cell density, where cell proliferation becomes impossible in overly-crowded regions. We have now added comments on neglecting mechanics to the manuscript (§2.2, p. 9, Lines 162–165, §4, p. 20, Lines 394–397).

3. Further to my comment above, why do the authors not model time? Is time-series data simply not available for these experiments? In which case, how can the authors be sure that the model-experiment comparison is drawn at the correct "time"?

   Response: Sparse time series data is available for these experiments (see the colony evolution in Figure 3). Inferring parameters from this time series data is a useful suggestion, which we will consider in future work. In this work, our intention is to use a simple model and parameter estimation method to understand colony morphology. Therefore, we only compare the simulation against the final experimental photograph. Since the computational cost of ABC is high, in practice we would need to use alternative parameter estimation methods, *e.g.* machine learning, to use the time series data. To ensure we compare the model with experiments at the correct time, we simulate colonies up to a prescribed cell number, such that the simulated colony has approximately the same area as the final experimental photograph. The physical time at which this occurs is unimportant, because the cell proliferation rate will not impact the colony morphology.

4. The authors should justify their choice of prior. The priors for p_{ps} and p_{sp} could reasonably be justified as being uninformative, however the same cannot be said for the other parameters. How do the results depend on prior choice? Why do the authors not consider a uniform distribution (i.e., Beta(1, 1))?

   Response: We have now added the following text justifying the choice of priors to the manuscript (§2.4, p. 15, Line, 298–309): "For two reasons, we chose weakly informative priors for n^*, γ, and p_a, and uninformative priors for p_{ps} and p_{sp}. The first reason is that physical intuition guides our expectation for the values these parameters may take, whereas less is known about p_{ps} and p_{sp}. We expect n^* to be relatively large, because experimental images show pseudohyphal growth emerging after a long initial period of circular (Eden-like sated) growth. The parameter p_a is likely to be small, because once the colony becomes starved of nutrients, we expect most of the growth to be pseudohyphal rather than sated. Finally, we expect the forking control mechanism controlled by γ to be invoked infrequently, because experimental images suggest that pseudohyphal branches do fork regularly. The second reason for using weakly informative priors is that they help the performance of the ABC parameter inference algorithm. ABC can become stuck if the chain enters regions of the parameter space where the simulated and experimental data are very different, and weakly informative priors can help prevent this."

   During the parameter inference, the MCMC chain would remain stationary if the choice of prior was too wide. For example, when n^* was set too small, the distance between the simulation and experimental images would be too far for the chain to converge. This is also why we initialised the parameter near the mean of the priors.

5. Did the authors consider performing a formal model-selection procedure for the three budding patterns proposed at the bottom of page 6?

   Response: We did not perform a formal model selection procedure for the cell half-width,

half-length, and budding angle. Although we cannot completely rule out the possibility of varying cell sizes and shapes, these parameters are well known based on experiments reported in the literature [2]. We neglected to include this reference in the table from the original manuscript, so this is now included (§2.2, p. 7, Table 1). We expect these parameters to remain consistent across strains and nutrient conditions (now mentioned in §2.2, p. 7, Lines 135–136), and hold them constant in the simulations. Fixing these parameters is useful to reduce the computational complexity of the model, which is important for the ABC inference given that we use few summary statistics. Given that sated and pseudohyphal cells with these dimensions have similar volume, fixing these parameters is also consistent with the idea that nutrient concentration varies linearly with cell number.

Minor Comments

1. The authors should provide justification (or otherwise, a sensitivity-type analysis) for the parameters chosen in Table 2.1.

   Response: As indicated above, the cell size parameters in Table 1 were based on the experimental measurements of Gimeno et al. [2], which we now reference (§2.2, p. 7, Table 1).

2. Do the authors expect any parameters to be identical between strains?

   Response: We assume that cell half-width, half-length, and budding angle are identical between strains (§2.2, p. 7, Lines 135–136). Less is known about the five parameters that we infer, so we do not assume these to be identical across strains. Since we had no biological intuition to distinguish these five parameters between strains, we used ABC to investigate similarities and differences.

3. The authors state that it is possible to infer parameters for single experimental images, but this could lead to over fitting. I would expect, rather, to just have possible identifiability issues. However, if the summary statistics are comprehensive enough and the variability in summary statistics is small enough between images, the posteriors should be similar.

   Response: With this statement, our intention was to communicate that fitting parameters to a single colony would not necessarily provide good posterior distributions for the parameters, because there is variability across the experiments. The term 'over-fitting' was therefore misleading. We apologise for the confusion, and have amended the manuscript (§2.4, p. 14, Lines 295–296). The model does not capture all potential sources of experimental variation. For example, the initial colony size cannot be determined from the experiments, so we initiate each simulation from a single cell. However, we believe that the variation across experiments arises due to randomness in colony growth and not the initial condition, so to obtain representative distributions for the parameters we infer parameters against the mean of the summary statistics.

Reviewer 2

The paper is interesting, the described methods are elegant. It communicates the motivation of the work, the applied methods and the results clearly. The text is also well written.

Response: We are grateful to Reviewer 2 for their careful reading of our manuscript and helpful suggestions. The changes we have made are outlined below, and printed in magenta within the paper for ease of identification.

Questions, Comments

1. What were the reasons behind using the two mentioned yeast strains?

   Response: The species *S. cerevisiae* has several advantages for experimental research. Its genome has been fully sequenced, and it is a readily available and non-toxic model organism for fungal growth. The particular strains used, AWRI 796 and Simi White, were chosen because of their relevance to wine research, but are also capable of producing different colony shapes in experiments (§1, pp. 2-3, Lines 26–28).

2. Figure 1.3: What were the experimental conditions (strain, nutrient level) of the colonies shown at the image?

   Response: We apologise for not including this information in the manuscript. The strain was AWRI 796 at $50\,\mu\mathrm{M}$. This information has now been included in the manuscript, (§1.1, p. 6, Line 100).

3. Figure 2.1 shows 5 budding sites at the end of the cell, while line 122 mentions 3 sites. Which value was used in the model?

   Response: The number of budding sites should be four. We have now amended this error in the text (§2.2, p. 8, Line 140). We have also updated Figure 2.1(a) by adding red dots to better indicate the potential budding sites (§2.2, p. 8, Line 140) at the angles $\pm\beta$ from the axial direction, and rewritten the text (§2.2, p. 8, Line 142–146) to improve clarity. We apologise for the initial confusion and thank the reviewer for their careful consideration.

4. Can filaments cross each other in the model? Would it possible or useful to extend the model into 3D?

   Response: It is possible, but unlikely, for branches of pseudohyphal cells to cross each other in the simulation. Given the volume exclusion constraint in the model, filaments crossing would be most likely when two filaments approach each other orthogonally. This is less likely for pseudohyphal cells, because they tend to grow radially away from the colony, with small budding angle β. This is also reflected in experimental images, where the filaments do not appear to cross frequently. Although crossing in the simulation may represent a 2D projection of 3D growth where a filament grows over another one, we do not believe this to be significant. We chose not to extend the model to 3D because cell growth is primarily confined to the 2D plane, and the available experimental data consists of 2D photographs.

5. What were the typical cell numbers in the simulations?

   Response: The average number of cells in each simulation is given below:

- AWRI 796 $50\mu M$: 37599 cells
- AWRI 796 $500\mu M$: 81810 cells
- Simi White $50\mu M$: 54908 cells

We have now reported this information in Table 1 of the Supporting Material.

6. What kinds of computational resources were used for the presented work? How much time a typical does a typical simulation required?

   Response: We used an Intel Xeon CPU E5-2699 v3 (2.30GHz) computer to perform the simulations. Typical simulation times for each strain/nutrient combination are given below:

   - AWRI 796 $50\mu M$: $171\,\mathrm{s}$
   - AWRI 796 $500\mu M$: $401\,\mathrm{s}$
   - Simi White $50\mu M$: $324\,\mathrm{s}$

   We have now reported this information in Table 1 of the Supporting Material.

7. Is there any cell death in the model? How could it influence the results?

   Response: Cell death is neglected in the model. This is a simplifying assumption, but is based on reported yeast cell lifespans of up to 10 days [5]. If cell death was included, the most death would occur among the oldest cells in the centre of the colony, which will have little effect on the filamentous morphology. Cell death might lead to further proliferation in the centre of the colony (to replace dead cells), which is prevented in our model by volume exclusion. In low-nutrient environments yeast cells can enter a stationary phase [6] instead of dying, where they remain dormant and may be able to begin to proliferate again if nutrient level is restored. We now comment on this in the manuscript (§2.2, pp. 8-9, Lines 156–158).

8. Line 222 states: "we only counted a quarter of the colony's branches". Could you elaborate on this (e.g.: which quarter was counted)?

   Response: We used the top left corner of the colony. In polar coordinates where the origin is the colony centroid, this represents the region (r, θ), for $r \in [r_{\mathrm{csr}}, r_{\max}]$, and $\theta \in [\pi/2, \pi]$. We now mention this in the manuscript (§2.3, p. 12, Lines 250–252).

9. How were the initial values of the parameters defined at the parameter inference?

   Response: The initial parameter values for parameter inference were chosen to be a small perturbation away from the mean of the prior distributions. We now mention this in the manuscript (§2.4, p. 15, Lines 314–315).

10. Figure 3.1: Were the differences between the showed metrics statistically significant?

    Response: We did not test whether the differences between the observed metrics in Figure 3.1 are statistically significant. Given that our objective was to use the combination of summary statistics to characterise colony morphology, it is not necessarily important for each experimental condition to yield a statistically significant difference for each individual summary statistic. It is possible to apply other summary statistics, *e.g.* pair correlation functions, but these were not needed for our purposes.

11. Could be any difference in the cell sizes, shapes or cell growths (division intensities) between the studied strains? Could these influence the differences between their colonies (on top of the studied parameters)?

    Response: Differences in cell size and shape or cell proliferation rate would impact the simulation results. Cell size and shape parameters are known (due to Gimeno et al. [2], now referenced in Table 1), and given that all experiments were performed with strains from the same family of *S. cerevisiae*, we assumed these to be constant across strains. In this work, we chose to focus on varying the five parameters $(n^*, p_a, p_{sp}, p_{ps}, \gamma)$ instead, because less is known about these. However, we cannot fully rule out the possibility of cell size and shape variation, and leave these as avenues for future research.

12. Link for the supporting materials is missing from the "Supporting Material" section. (It appears at the end of the manuscript.)

    Response: The Supporting Material will be provided separately as part of the finalised submission process — we trust that the correct link will be posted under the relevant heading once the published version of the paper becomes available.

13. Most of the references are older. Are there more state of the art publications on the topic?

    Response: Although the original reference describing pseudohyphal growth in yeast colonies by Gimeno et al. [2] was published more than 30 years ago, it represents the current view of how yeast colonies respond to low nutrient environments. To reinforce this, we have now included references to recent review articles on *S. cerevisiae* [4, 7] that reflect this view.

Minor Suggestions

1. Line 3: Yeasts (typo).
2. Line 144: Figure 1.3(g).

   Response: We thank the referee for pointing out these typographical and grammatical errors, which are now fixed in the revised manuscript.

3. I_B is both called branch count and sub-branch index.

   Response: The correct term for I_B is 'sub-branch count', whereas the term 'sub-branch index' referred to the normalised sub-branch count. We recognise that the distinction between raw and normalised summary statistics was not clear in the original manuscript. Therefore, we have updated the notation for the summary statistics in the manuscript. We apologise for the confusion and thank the reviewer for their detailed attention.

4. Line 284: means, deviations, statistics.
5. Figure 3.1: concentrations, strains.
6. Figure 3.2: statistics, areas.
7. Line 328: is in the.

   Response: We thank the referee for pointing out these typographical and grammatical errors, which are now fixed in the revised manuscript.

8. Line 376: Credit (capital letters).

   Response: The author contribution statement was intended to comply with the CRediT contributer roles taxonomy used by *PLoS Comput. Biol.* We have now amended the text underneath and apologise for the confusion.

References

[1] A. Tam, J. E. F. Green, S. Balasuriya, E. L. Tek, J. M. Gardner, J. F. Sundstrom, V. Jiranek, and B. J. Binder, "Nutrient-Limited Growth with Non-Linear Cell Diffusion as a Mechanism for Floral Pattern Formation in Yeast Biofilms", *Journal of Theoretical Biology* 448 (2018), pp. 122–141.

[2] C. J. Gimeno, P. O. Ljungdahl, C. A. Styles, and G. R. Fink, "Unipolar cell divisions in the yeast S. cerevisiae lead to filamentous growth: regulation by starvation and RAS", *Cell* 68 (1992), pp. 1077–1090.

[3] H. Tronnolone, A. Tam, Z. Szenczi, J. Green, S. Balasuriya, E. L. Tek, J. M. Gardner, J. F. Sundstrom, V. Jiranek, S. G. Oliver, et al., "Diffusion-limited growth of microbial colonies", *Scientific Reports* 8 (2018), 5992.

[4] A. Kumar, "The complex genetic basis and multilayered regulatory conrol of yeast pseudohyphal growth", *Annual Review of Genetics* 55 (2021), pp. 1–21.

[5] N. Minois, M. Frajnt, C. Wilson, and J. W. Vaupel, "Advances in measuring lifespan in the yeast Saccharomyces cerevisiae", *Proceedings of the National Academy of Sciences* 102 (2005), pp. 402–406.

[6] M. Werner-Washburne, E. L. Braun, M. E. Crawford, and V. M. Peck, "Stationary phase in *Saccharomyces cerevisiae*", *Molecular Microbiology* 19 (1996), pp. 1159–1166.

[7] R. Abid, H. Waseem, J. Ali, S. Ghazanfar, G. Muhammad Ali, A. M. Elasbali, and S. H. Alharethi, "Probiotic yeast Saccharomyces: Back to nature to improve human health", *Journal of Fungi* 8 (2022), p. 444.
