# Supplementary material for: An off-lattice discrete model to characterise filamentous yeast colony morphology
Source: PLoS Comput Biol. 2024 Nov 21;20(11):e1012605. doi: 10.1371/journal.pcbi.1012605 (PMC11620580; doi:10.1371/journal.pcbi.1012605)
Supplement: Attachment — Submitted filename: responses.pdf [file pcbi.1012605.s004.pdf]

Response to Reviewers

An off-lattice discrete model to characterise filamentous yeast colony morphology

Kai Li, J. Edward F. Green, Hayden Tronnolone, Alexander K. Y. Tam, Andrew J. Black, Jennifer M. Gardner, Joanna F. Sundstrom, Vladimir Jiranek, Benjamin J. Binder

Reviewer 1

I thank the authors for responding to each of my original comments. While I do find many of the responses reasonable, I feel that none of my original concerns were actually properly addressed (but rather, rebutted). The comments, in particular, that I feel need to be addressed are as follows.

Response: We thank the reviewer for their time with this revision. Please see below for our responses to each of the queries.

1. The authors claim very early on that "initial nutrient concentration" among other features, was a key determining feature that determines the shape and structure of the colony, however they do not explicitly model nutrients. They do have experimental information that could give insight into this (two experiments with the same strain, with a 10-fold difference in nutrient concentration), with which they could do a statistical test (for instance) to determine if key parameters are different. How do the authors reconcile the 10-fold difference in nutrient concentration with similar values of n^* (as per the posterior distributions in Fig. 8)? Presumably, the authors could, for instance, use a fit to AWRI 50 µM data to test their hypothesis that nutrient concentration is proportional to cell-count to predict AWRI 500 µM data.

   In short, I am of the view that the authors cannot state any conclusions relating to nutrient concentration without either: (1) performing some kind of statistical demonstration to discuss how n^* is different between 50 µM and 500 µM measurements; (2) performing some kind of validation for the nutrient-cell count assumption; or (3) explicitly modelling the nutrient concentration.

   Response: We thank the reviewer for this comment, and acknowledge that our description of the impact of nutrient level on colony morphology was unclear. We acknowledge that attempting to relate the model parameter n^* with the nutrient level based on nutrient consumption being proportional to cell count was an overly simplistic view, and apologise for the confusion caused. To address this issue, we have rewritten the parts of the paper that interpret the parameter n^*. In the abstract, we now refer to this n^* parameter as the 'colony size at the transition from sated to pseudohyphal growth', and this will be

impacted by environmental stressors. The next major change was to remove discussion relating the n^* to nutrient concentration in Section 2.2, which appeared in lines 137–139, 169–172, and 178–188 in the previous version (see Lines 138–139, 167–168, 174–176, 180 in the newly-revised version). This has been replaced with a discussion about the inferred values of n^* for the $50\,\mu\text{M}$ and $500\,\mu\text{M}$ colonies (Section 3.2, Lines 332–338). Using the inferred values of n^* and $n_{\max}$, there were $0.67 \times 37599 = 25,191$ cells and $0.81 \times 81810 = 66,266$ cells before the sated-to-pseudohyphal transition, in the AWRI $50\,\mu\text{M}$ and AWRI $500\,\mu\text{M}$ colonies, respectively. Although there is an earlier transition to pseudohyphal growth in low nutrient environments as expected, the cell number is not proportional to the nutrient level. A possible explanation is that sustaining a larger colony requires more nutrients, such that larger colonies have a higher critical nutrient threshold than smaller colonies. However, we now refrain from directly linking nutrient level to n^*, because we have no experimental measurements for potential environmental stressors, such as nutrient level or toxins, as the colony grows. The lack of experimental data was also the reason for our decision to not model the nutrient concentration explicitly. We now believe that the current version of the manuscript correctly interprets the results relating to n^*.

2. I accept that ABC has known convergence issues, however the parameter space is relatively small (only five parameters). For example, ABC MCMC or even ABC SMC can give good results even for relatively uninformative priors. Investigating the effect of prior choice on at least one of the conditions would significantly strengthen the paper.

   Response: We thank the reviewer for this suggestion. We have investigated the effect of prior choice for the AWRI 796 $50\,\mu\text{M}$ colonies with uniform priors instead of weakly informative priors, as shown in Figure 1. This run took around 222 hours to complete. The reported mean values in Fig. 10 were $\theta = (n^*, p_a, p_{sp}, p_{ps}, \gamma) = (0.67, 0.14, 0.25, 0.58, 0.12)$. In comparison, the mean values found using uniform priors were $\theta = (n^*, p_a, p_{sp}, p_{ps}, \gamma) = (0.61, 0.11, 0.22, 0.61, 0.12)$. The comparison shows our choice of priors does not make a large difference to the resulting posteriors. We have commented on this in the manuscript on Lines 302-305 and included the traceplot below in Supplementary Material S3.

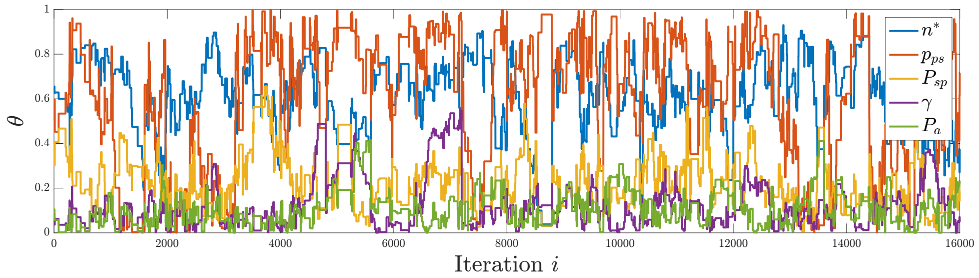

Figure 1: Traceplot of ABC-MCMC algorithm for AWRI 796 $50\,\mu\text{M}$ using uniform uninformative priors.

3. (relatively minor) In response to original minor comment 3, the authors state that they initialise the colonies from a single cell. Is this actually how the experiments are initiated? This could significantly impact the effect of the nutrient-cell count model, since a colony that is initiated with a set nutrient count at a size that is not zero would have additional biomass not accounted for by nutrient consumption.

Response: It is correct that the simulations were initialised with a single cell, and that this does not directly correspond to the experiments. In experiments, a small droplet containing cells is placed in the Petri dish, but we do not know the precise initial cell number. As the reviewer indicates, the difference in initial conditions between the experiments and simulation is another factor complicating attempts to relate n^* with the nutrient level. This is another reason that we needed to reinterpret the results as discussed for Comment 1.
